# Optical Redox Imaging of Treatment Responses to Nampt Inhibition and Combination Therapy in Triple-Negative Breast Cancer Cells

**DOI:** 10.3390/ijms22115563

**Published:** 2021-05-25

**Authors:** Allison Podsednik, Jinxia Jiang, Annemarie Jacob, Lin Z. Li, He N. Xu

**Affiliations:** Britton Chance Laboratory of Redox Imaging, Department of Radiology, Perelman School of Medicine, University of Pennsylvania, Philadelphia, PA 19104, USA; podsednik@alumni.upenn.edu (A.P.); jinjiang@sas.upenn.edu (J.J.); jacobann@seas.upenn.edu (A.J.); linli@pennmedicine.upenn.edu (L.Z.L.)

**Keywords:** NADH and flavoproteins containing FAD, redox ratio, FK866/APO866, paclitaxel/Taxol, nicotinamide riboside, FX11, ROS

## Abstract

We evaluated the utility of optical redox imaging (ORI) to identify the therapeutic response of triple-negative breast cancers (TNBC) under various drug treatments. Cultured HCC1806 and MDA-MB-231 cells treated with FK866 (nicotinamide phosphoribosyltransferase (Nampt) inhibitor), FX11 (lactate dehydrogenase A inhibitor), paclitaxel, and their combinations were subjected to ORI, followed by imaging fluorescently labeled reactive oxygen species (ROS). Cell growth inhibition was measured by a cell viability assay. We found that both cell lines experienced significant NADH decrease and redox ratio (Fp/(NADH+Fp)) increase due to FK866 treatment; however, HCC1806 was much more responsive than MDA-MB-231. We further studied HCC1806 with the main findings: (i) nicotinamide riboside (NR) partially restored NADH in FK866-treated cells; (ii) FX11 induced an over 3-fold NADH increase in FK866 or FK866+NR pretreated cells; (iii) FK866 combined with paclitaxel caused synergistic increases in both Fp and the redox ratio; (iv) FK866 sensitized cells to paclitaxel treatments, which agrees with the redox changes detected by ORI; (v) Fp and the redox ratio positively correlated with cell growth inhibition; and (vi) Fp and NADH positively correlated with ROS level. Our study supports the utility of ORI for detecting the treatment responses of TNBC to Nampt inhibition and the sensitization effects on standard chemotherapeutics.

## 1. Introduction

Breast cancer is the most diagnosed cancer among women, with ~15% of breast cancer patients possessing a triple-negative breast cancer (TNBC) subtype, i.e., absence of estrogen and progesterone receptors (ER^−^, PR^−^), and lack of HER2 overexpression (HER2^−^) [1,2]. With current treatment options limited to surgery and systemic chemotherapy, TNBC has the worst prognosis among breast cancer molecular types (https://www.breastcancer.org/symptoms/types/molecular-subtypes, accessed on 21 May 2021). TNBC is also a highly heterogeneous group of breast cancers with diverse therapeutic responses to chemotherapy [3,4]. Sensitive and early biomarkers for response to chemotherapy are crucial for the determination of responders versus non-responders and optimization of cancer treatment strategies [5]. 

Metabolism has been at the center stage of cancer research in recent decades. On one hand, metabolic changes at the molecular level precede morphological/pathological changes and are expected to provide early biomarkers for treatment response. On the other hand, cancer metabolism provides new therapeutic targets that will potentially enhance treatment effects when combined with conventional chemotherapy. For example, there has been a renewed interest in nicotinamide adenine dinucleotide (NAD^+^) biology and targeting enzymes involved in NAD^+^ metabolism has been proposed as a cancer therapy [6,7]. NAD^+^, an essential molecule for cellular metabolism, plays a central role in mitochondrial energy transduction and is synthesized by two major pathways: de novo and salvage pathways [8,9]. Nicotinamide phosphoribosyltransferase (Nampt), the key and rate-limiting enzyme of the salvage pathway of NAD^+^ biosynthesis, is crucial for the maintenance of intracellular NAD^+^ levels and regulation of NAD-dependent enzymes. Data from clinical breast cancer patients and many breast cancer cell lines show overexpression of Nampt [10,11]. While deregulation of Nampt expression is related to initiation and progression of various human malignancies [12], Nampt inhibition has led to tumor growth attenuation in various cancers [6,13]. Furthermore, suppression of Nampt expression has been found to reduce the viability of breast cancer cells and increase their susceptibility to chemotherapy [12,14]. 

Optical redox imaging (ORI, or optical metabolic imaging, used in some literature) is a label-free metabolic imaging technique that may aid in the development of cancer biomarkers for treatment response. It detects the intrinsic fluorescence of oxidized flavoproteins (Fp containing flavin adenine dinucleotide (FAD)) and reduced nicotinamide adenine dinucleotide (NADH) [15,16,17]. The optical redox ratio, Fp/NADH or its normalized form Fp/(NADH+Fp), is a surrogate marker of the NAD^+^/NADH or NAD^+^/(NADH+NAD^+^) ratios, respectively, and provides a quantitative measure of the mitochondrial redox state. It has been shown that Fp/NADH or Fp/(NADH+Fp) linearly correlates with biochemically-determined redox ratio NAD^+^/NADH or NAD^+^/(NADH+NAD^+^) [18,19,20]. ORI has wide applications in the study of bioenergetics, metabolism, and treatment response [21,22,23]. Employing ORI, we previously demonstrated frontline therapy CHOP-induced mitochondrial redox state alteration in non-Hodgkin’s lymphoma xenografts [24]. We also reported that lonidamine-induced redox changes in melanoma were readily detected by ORI at both cellular and tissue levels within 45 min after treatment [25], in accordance with lonidamine’s anti-tumor effect and metabolic changes detected by ^31^P-MRS in the same melanoma xenograft models [26,27,28]. Based on NADH and FAD intensity and lifetime measurements, multiphoton optical metabolic imaging has also been used to identify early therapeutic responses of various cancer cells and differentiate between drug-resistant and non-resistant models [29,30,31,32], and was able to resolve treatment response in tumor xenografts earlier than fluorodeoxyglucose positron emission tomography (FDG-PET) [29].

Herein, we examine the utility of ORI for detecting the therapeutic response to Nampt inhibitor FK866, a common chemo-agent paclitaxel (Taxol), and their combinations, using two TNBC cell culture models that have different sensitivities to Nampt inhibition. We correlate the ORI readouts with an MTS (3-(4,5-dimethylthiazol-2-yl)-5-(3-carboxymethoxyphenyl)-2-(4-sulfophenyl)-2H-tetrazolium) cell viability assay, which acts as the endpoint for the treatment responses. We also investigate the correlation of ORI indices with the intracellular levels of reactive oxygen species (ROS).

## 2. Results

### 2.1. Redox Responses to FK866 Treatment and NR Rescue Effects 

**Figure 1:**
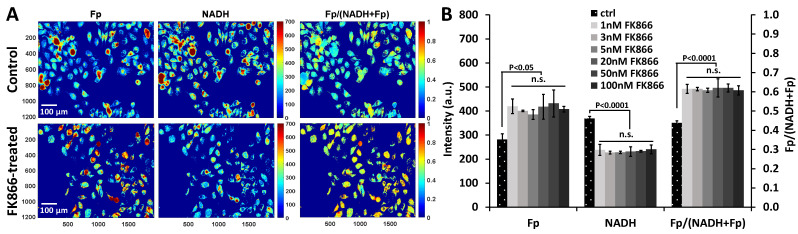
Redox responses to FK866 in HCC1806 cells. (**A**) Typical pseudo-colored images of HCC1806 cells treated with 0.1% DMSO (control) or 1 nM FK866 for 48 h. X and Y coordinates are shown in the redox images. For both Fp and NADH images, the color bars represent signal intensity in arbitrary units ranging from 0 to 700. For the Fp/(NADH+Fp) images, the color bars represent the redox ratio ranging from 0 to 1. (**B**) Quantification of Fp and NADH intensities and the redox ratios of HCC1806 cells with various concentrations of FK866, ranging from 1 to 100 nM (*n* = 3 for each FK866 concentration).

FK866 is expected to decrease NAD^+^ and its protonated/reduced form, NADH. ORI was applied to detect the response to 48 h FK866 treatment in two TNBC breast cancer cell lines, HCC1806 and MDA-MB-231. Figure 1A shows the typical redox images of HCC1806 cells under the control condition (0.1% DMSO) and 1 nM FK866 treatment. Treatment with various concentrations of FK866 ranging from 1 nM (the lowest concentration we tested) to 100 nM for 48 h generated similar effects on the redox indices (Fp, NADH, and the redox ratio) and significantly increased Fp signals by ~49%, decreased NADH signals by ~35%, and raised the redox ratio by ~40% (Figure 1B). FK866-induced Fp increase coincided with NADH decrease, which likely reflects the conjugation of the two signals in the mitochondria. 

The phenomenon that 1 to 100 nM FK866 yielded the same redox effect indicates that in this range the NAD-salvage pathway has been completely inhibited at FK866 concentration as low as 1 nM. This apparent saturation effect can be further understood by the fact that in many cell lines, the IC_50_ of FK866 is below 1 nM [33], and many companies report IC_50_ is 0.09 nM (e.g., https://www.selleckchem.com/products/apo866-fk866.html, accessed on 21 May 2021). 

**Figure 2:**
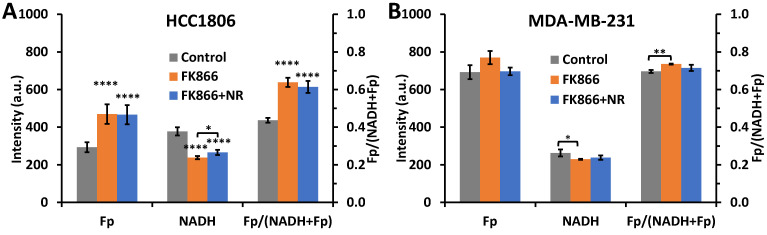
NR effects on the redox indices in TNBC cells. (**A**) HCC1806 cells (*n* = 6). (**B**) MDA-MB-231 cells (*n* = 3); FK866 group: 100 nM FK866 48-h treatment; FK866+NR group: 100 nM FK866 48-h treatment during which 800 µM NR was added in the last 6 h. Stars above bars represent significant difference from control and stars above brackets indicate significant difference between treatment groups (ANOVA with Tukey’s post-hoc test, * *p* < 0.05, ** *p* < 0.01, **** *p* < 0.0001).

Nicotinamide riboside (NR) is a precursor of NAD^+^. NR is converted to nicotinamide mononucleotide (NMN) by an NR kinase (NRK), then NMN is converted to NAD^+^ by NMN adenylyltransferase (NMNAT) [34]. By adding NR to cells pre-treated with FK866, which have diminished use of the NAD-salvage pathway, we expected to see an increase in NAD^+^ and thus NADH. We added NR (800 µM) to the dishes pre-treated with 100 nM FK866 for 42 h. Six hours later, we found an 11.7% increase in NADH (*p* = 0.02) in HCC1806 cells compared to the NADH level of 100 nM FK866 48 h treated HCC1806 dishes (Figure 2A). Though NR treatment significantly increased the NADH signal from FK866 pre-treated HCC1806 cells, the drug at the concentration of 800 µM did not fully restore NADH levels. Furthermore, the NR rescue effect was nearly equivalent in the various concentrations of FK866 tested (5–100 nM) (Figure S1). This result further supports that the NAD-salvage pathway has been completely inhibited at 1 nM of FK866.

For the MDA-MB-231 cells, 48 h treatment with 100 nM FK866 also resulted in a significant but lesser degree of NADH decrease and redox ratio increase but no significant change in Fp (Figure 2B). This result is consistent with literature reports that the MDA-MB-231 cell line is relatively less sensitive to Nampt inhibition [11]. In contrast to HCC1806 cells, no effects by NR were found in MDA-MB-231 cells, which could also serve as a negative control. We focused on studying HCC1806 cells only in the following experiments.

### 2.2. Inhibiting Lactate Dehydrogenase A of FK866-Pretreated Cells Resulted in a Dramatic NADH Spike

**Figure 3:**
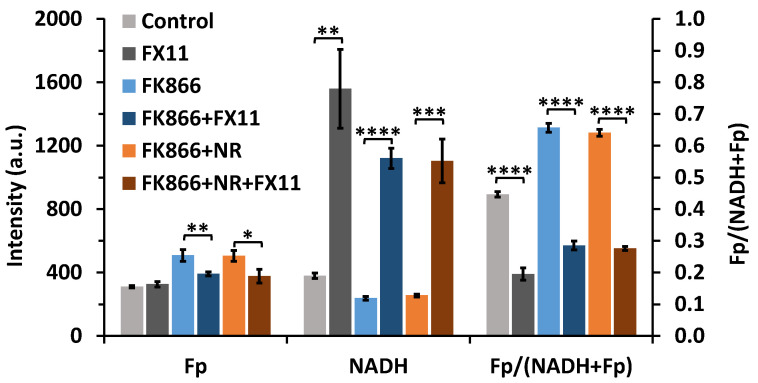
Redox effects of 10-min FX11 treatment on HCC1806 cells (*n* = 3). A total of 5 µM FX11 was added to control cells (0.1% DMSO, 48 h), cells pre-treated with 48 h 100 nM FK866, and cells pre-treated with 48 h 100 nM FK866 + 6 h 800 µM NR (unpaired *t*-test, * *p* < 0.05, ** *p* < 0.01, *** *p* < 0.001, **** *p* < 0.0001).

It is generally understood that Nampt regulates NAD^+^ synthesis and NADH pool size. For Nampt inhibition with long-term (48 h) FK866 treatment, we expect the availability of NADH should be very low if not diminished. Thus, we set out to investigate the NADH availability by adding FX11, a specific inhibitor of lactate dehydrogenase A (LDHA), to the HCC1806 cells that were pretreated with FK866 or FK866+NR. LDHA catalyzes the conversion of pyruvate to lactate, coupled with the oxidation of NADH to NAD^+^ in the cell. As expected, FX11 treatment to control dishes resulted in LDHA inhibition and a buildup of NADH (Figure 3, bars in grey colors), which was also previously observed [35]. However, to our surprise, after suppression of NADH levels by 100 nM FK866 for 48 h, the HCC1806 cells still responded strongly to 10 min 5 µM FX11 treatment, resulting in a dramatic increase in NADH, together with a significant decrease in Fp and the redox ratio (Figure 3, bars in blue colors). Cells pre-treated with FK866+NR also showed a similar response to FX11 treatment (Figure 3, bars in orange colors). The NADH increases for the control, FK866, and FK866+NR dishes are 1180, 884, and 848 units, corresponding to an increase of 311%, 372%, and 332%, respectively. These data suggest that a significant amount of NADH remains available despite the inhibition of the NAD^+^ salvage pathway.

### 2.3. ORI-Detected Therapeutic Responses Correlated with Growth Inhibition 

**Figure 4:**
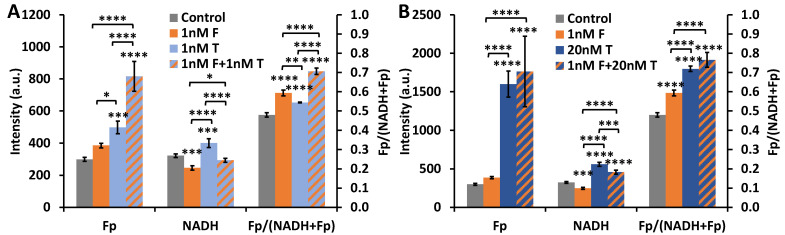
ORI indices respond to individual and combination treatment of FK866 and paclitaxel (48 h, *n* = 3). (**A**) 1 nM FK866 and 1 nM paclitaxel; (**B**) 1 nM FK866 and 20 nM paclitaxel. Control dishes for both graphs were treated with 0.2% DMSO. F stands for FK866 and T for paclitaxel/Taxol. Stars above bars represent significant difference from control. Stars above brackets indicate significant difference between treatment groups (ANOVA with Tukey’s post-hoc test, * *p* < 0.05, ** *p* < 0.01, *** *p* < 0.001, **** *p* < 0.0001).

Paclitaxel is a chemotherapeutic agent for treating solid tumors, including breast tumors. We investigated paclitaxel treatment effects and whether a low concentration of FK866 may sensitize cells to paclitaxel treatment. Our ORI results illustrate that 1 nM paclitaxel (the reported IC_50_ for HCC1806 cells for 48 h treatment was in this range [36,37]) alone induced an increase in all redox indices (Figure 4A). Separate 1 nM FK866 and 1 nM paclitaxel treatments induced a total 96% increase in Fp level, and a summed change of 37% in the redox ratio. However, when cells were simultaneously treated with 1 nM FK866 and 1 nM paclitaxel, Fp levels increased by 173%, and the redox ratio increased by 47%, implying a synergistic effect on the redox status. Since FK866-induced NADH change was opposing the direction of the paclitaxel-induced change, we observed a lesser increase in NADH from the combination compared to that by paclitaxel alone. 

When we increased the paclitaxel concentration from 1 to 20 nM, Fp level was 435% higher than the control, which was triple the Fp level with 1 nM paclitaxel treatment; NADH level and the redox ratio were 73% and 50% higher than the control, respectively, corresponding to 39% and 32% higher than that from 1 nM paclitaxel treatment, respectively (Figure 4B). The individual Fp percentage increase for 1 nM FK866 or 20 nM paclitaxel treatment summed to 464%, whereas the percentage increase in Fp for simultaneous treatment at these concentrations was 490%. Thus, the synergistic effect on Fp for the combination 1 nM FK866 + 20 nM paclitaxel treatment was modest and less prominent than that of 1 nM FK866 + 1 nM paclitaxel. The 42% NADH change due to 1 nM FK866 + 20 nM paclitaxel combination treatment was significantly different in comparison with either 1 nM FK866 (−24%) or 20 nM paclitaxel (73%) alone. No synergy on the redox ratio was present for 1 nM FK866 + 20 nM paclitaxel treatment despite that the largest redox ratio change in comparison with control was observed with the combination 1 nM FK866 + 20 nM paclitaxel treatment.

**Figure 5:**
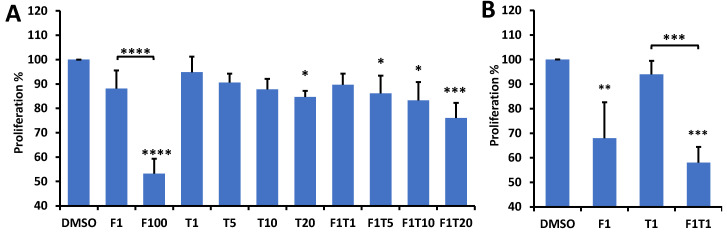
Effects of 48 h treatment with FK866, paclitaxel, and combinations of both on cell proliferation measured by MTS assay. In the x-axis, F represents FK866, T represents paclitaxel, and numbers following either F or T indicate concentration in nM. Stars above bars indicate significant difference from DMSO (control). Stars above brackets indicate significant difference between treatment groups. Means were normalized to DMSO mean. Significance determined by running one-way ANOVA with control for multiple comparisons. (**A**) shows the results with cell seeding density at 15,000/well (*n* = 3); (**B**) shows the results with cell seeding density at 5000/well (*n* = 4) (ANOVA with Dunnett’s post-hoc test, * *p* < 0.05, ** *p* < 0.01, *** *p* < 0.001, **** *p* < 0.0001).

With 48-h treatments of FK866 and paclitaxel on HCC1806 cells at various concentrations, the MTS assay revealed that FK866 and paclitaxel combination treatments inhibited cell proliferation (Figure 5). Specifically, from Figure 5A, HCC1806 cells exhibited a trend of decreased proliferation with 48 h 1 nM FK866 treatment and a significantly decreased proliferation by ~40% with 100 nM treatment. As for paclitaxel treatment alone, only the 20 nM dose resulted in a modest but significant proliferation inhibition. Combinations of 1 nM FK866 with 5, 10, and 20 nM paclitaxel significantly reduced proliferation, although no synergistic reduction was observed for the combinational treatment compared to individual treatments. We further confirmed this result by using a lower seeding density of cells/well. As displayed in Figure 5B, 1 nM FK866 treatment for 48 h showed a significant growth inhibition, as did the combination of 1 nM FK866 and 1 nM paclitaxel. However, 1 nM FK866 failed to show synergy with 1 nM paclitaxel treatment.

**Figure 6:**
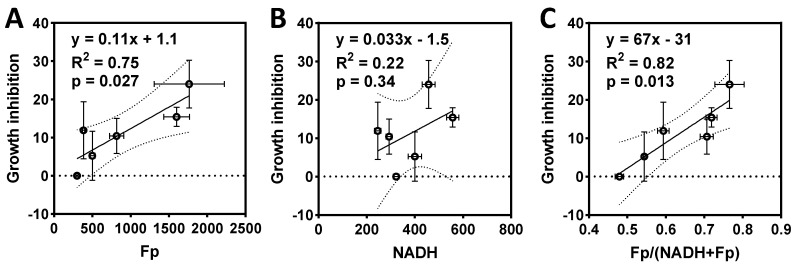
Correlations between the redox indices and growth inhibition of HCC1806 cells treated with FK866, paclitaxel, or the combinations (DMSO, F1, T1, T20, F1T1, F1T20). (**A**) Fp positively correlates with cell growth inhibition with the linear fit equation, correlation coefficient R^2^, and *p* value shown; the dashed curves represent the 95% confidence band. (**B**) No significant correlation was found between NADH and growth inhibition; (**C**) the redox ratio positively correlates with growth inhibition.

Comparing the results from ORI and MTS (Figure 4 and Figure 5) indicates that the ORI-detected redox changes correspond to the MTS-detected proliferation inhibition of HCC1806 cells, and larger redox ratio change corresponds to larger inhibition of proliferation. However, ORI appears to be more sensitive in detecting both single-treatment effect and sensitization effect. Particularly, at a low concentration of drugs, synergistic redox changes induced by these treatments were observed, whereas MTS did not detect such synergy. We performed a linear regression analysis based on the data from Figure 4 and Figure 5A (DMSO, F1, T1, T20, F1T1, F1T20) and found both Fp and the redox ratio positively correlate with growth inhibition, whereas NADH does not (Figure 6). The significant linear correlation indicates that either Fp or the redox ratio can predict treatment responses to FK866, paclitaxel, and their combination in HCC1806 cells.

**Figure 7:**
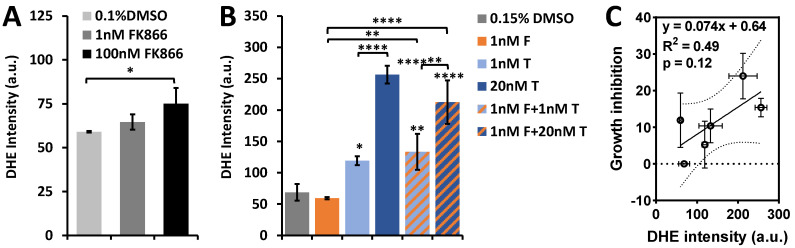
Intracellular ROS levels in HCC1806 cells under 48 h FK866 and/or paclitaxel treatment. (**A**) A trend of increasing intracellular ROS generation with increasing FK866 concentration (*n* = 3). (**B**) Intracellular ROS generation in response to FK866 (F), paclitaxel (T), and combinations of both agents (*n* = 3). Stars above bars represent significant difference from control (DMSO) and stars above brackets indicate significant difference between treatment groups. (**C**) No significant linear correlation between ROS generation and growth inhibition as shown by the *p* value. Dashed lines represent the 95% confidence band (ANOVA with Tukey’s post-hoc test, * *p* < 0.05, ** *p* < 0.01, **** *p* < 0.0001).

We previously found that the redox indices correlate with ROS levels [35]. We therefore also measured drug-induced intracellular ROS production. As shown by Figure 7A, FK866 treatment led to a modest increase in intracellular ROS level, which was insignificant at 1nM, yet ~27% significantly increased at 100 nM. In contrast, there was a ~73% ROS increase due to 1 nM paclitaxel treatment and a 94% ROS increase due to combination of 1 nM FK866 and 1 nM paclitaxel treatment compared to control. Additionally, there were larger significant increases in ROS level due to 20 nM paclitaxel treatment (~273%) and combination of 1 nM FK866 and 20 nM paclitaxel treatment (~209%) (Figure 7B). These findings are consistent with literature reports [11,38,39,40,41]. However, the correlation between growth inhibition and ROS is not statistically significant (Figure 7C). Note that 1 nM FK866 addition did not significantly increase ROS generation in the cells that were under 1 or 20 nM paclitaxel treatment. In comparison, ORI detected a significant redox difference under these conditions. 

**Figure 8:**
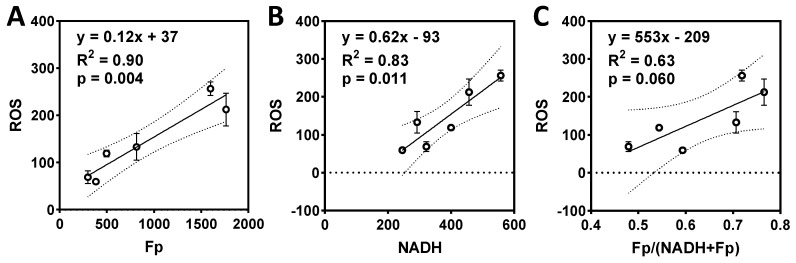
The correlations between the redox indices and the drug-induced intracellular ROS of HCC1806 cells treated with FK866, paclitaxel, or the combinations (DMSO, F1, T1, T20, F1T1, F1T20). (**A**) Fp positively correlates with ROS level; (**B**) NADH positively correlates with ROS level; (**C**) there is a positive trend of correlation between the redox ratio and ROS level. Dashed lines represent the 95% confidence band.

We further performed a linear regression analysis to determine the correlation between the redox indices and ROS levels under these various treatments. As shown in Figure 8, both Fp and NADH positively correlate with ROS (*p* = 0.004 and 0.011, respectively) (Figure 8A,B). Although higher redox ratio tends to correspond to higher ROS, the correlation has a borderline significance (*p* = 0.060).

## 3. Discussion

### 3.1. ORI Is Sensitive to the Metabolic Modulations and Detects Differential Responses to Nampt Inhibition between Two TNBC Cell Lines

Optical redox imaging of NAD(H) redox status provides a measure of the mitochondrial redox status and has been found to be sensitive to the therapeutic effects of cancer drugs. Here, we employed ORI to investigate the effects of inhibition of NAD biosynthesis by treating TNBC cells with Nampt inhibitor, FK866. We observed significant redox changes in both HCC1806 and MDA-MB-231 cells. We found that NAD(H) redox status responded differentially in these two TNBC lines treated with 100 nM FK866 for 48 h, where NADH decreased by ~40% and ~13% in HCC1806 and MDA-MB-231 cells, respectively. Since several factors, including timing, the FK866 dose, and the 3-phosphoglycerate dehydrogenase (PHGDH) expression level can all affect NAD^+^ depletion [11,42], NADH level should also be affected by these factors. The temporal NAD^+^ depletion pattern shows that with 10 nM FK866 treatment, the NAD^+^ level of MDA-MB-231 cells reached the lowest at 24 h then went up at 48 h [11], suggesting we could have observed lower NADH levels at 24 h instead of at 48 h. On the other hand, Nampt inhibition affects serine biosynthesis from glucose via PHGDH, and the PHGDH-high breast cancer cell lines (estrogen receptor absence, basal-like, such as HCC1806) are highly sensitive to Nampt inhibition compared to PHGDH-low cell lines (estrogen receptor absence, mesenchymal, such as MDA-MB-231) [11,42]. These factors together may explain a smaller redox change in MDA-MB-231 than in HCC1806 upon 48 h FK866 treatment with the same dose.

Biochemical assay analysis has shown that suppression of Nampt in breast cancer cells lowers NAD^+^, NADH, and NADPH levels, where NADH decrease is less than NAD^+^ decrease [38,39,43]. By treating the two TNBC cell lines with a Nampt inhibitor, FK866, for 48 h, we found a significant decrease in NADH level, consistent with these reports. We also found an FK866-induced increase in Fp. Thus, both a decreased NADH and an increased Fp contribute to an increase in the redox ratio in the TNBC cells. Moreover, the FK866 in the range of 1 to 100 nM had an equivalent impact on the redox status of HCC1806 cells. This suggests that lower FK866 concentrations would have had impacts on the NAD(H) redox status of HCC1806 cells as well. It also warrants further testing with higher FK866 concentrations, since a concentration-dependent effect of FK866 on NAD^+^ level has been reported for many types of cells [44].

By modulating the HCC1806 cells with NR, we observed the expected redox changes. NR increased NADH, although NADH was not fully restored to its original level. The NADH rescue effect by NAD^+^ supplementation depends on several factors, including specific NAD^+^ restoration agents and their concentrations, as well as FK866 concentration used for NAD^+^ suppression [11]. 

It is known that TNBC cells are highly heterogeneous, resulting in diverse treatment outcomes. Therapeutic response biomarkers that are sensitive and that can stratify TNBC patients are highly desired. The observed differential redox responses to Nampt inhibition and NR restoration between the two TNBC lines suggest that ORI can be useful to improve classifications of TNBCs based on their redox responses to metabolic treatments. 

We also probed the NADH pool in HCC1806 cells by using FX11 to inhibit LDHA under the control or the pretreatment of FK866 or FK866+NR. Immediately after FX11 was added to the control or the FK866-treated HCC1806 cells (with or without NR rescue), we observed dramatic NADH increases, more than three times higher, and an over 50% decrease in redox ratios, compared to the levels before FX11 addition. Previously, we reported a ~200% NADH increase in MDA-MB-231 cells induced by FX11 treatment [35]. These results demonstrate the significant role of LDHA in mediating the NAD(H) redox balance in TNBC cells. This also seems to suggest that separate subcellular pools of NAD^+^ may be acted on by NR and FX11. It was reported that there is a mitochondrial-insensitive NAD^+^ pool and that FK866 reduces the cytoplasmic but not the mitochondrial NAD^+^ pool, which could be due to a delay in depletion of mitochondrial NAD^+^ [44]. It was also suggested that the NAD^+^ pool generated by Nampt exists as a separate pool to the NAD^+^ pool for glycolysis [11].

### 3.2. ORI Detects Paclitaxel Treatment Response and the Sensitization Effect of FK866 on Paclitaxel

We observed paclitaxel-induced dose-dependent redox changes, including increased NADH and Fp levels and the redox ratio, where both Fp and NADH increases correlated with drug-induced ROS production. The increase in NADH is likely the result of cells being apoptotic. It has been shown that when apoptosis starts, there is a significant increase in NADH signals, whereas H_2_O_2_-induced necrosis showed a decrease in NADH [45,46,47]. Lukina et al. reported paclitaxel-induced redox changes of 3D HeLa culture, where cells underwent a time-dependent increase in the optical redox ratio, starting from 6 h of exposure to paclitaxel [31]. However, their study observed no change in Fp intensity for any occasion, a decrease in NADH intensity in the responders (viable and altered morphology), and no NADH intensity change in non-responders (viable and unaltered morphology) within 24 h of treatment. It is unclear why our results of increased NADH and Fp levels after 48 h treatment differ from theirs. It could be due to different cancer cell types and/or treatment time. 

We observed that the combination of FK866 (1 nM) and paclitaxel (1 and 20 nM) resulted in synergistic redox changes in HCC1806 cells, corresponding to enhanced inhibition of cell growth detected by the MTS assay. A combination of Nampt inhibitors (including FK866) and paclitaxel has been shown to have an additive effect on decreasing cell viability and growth in pancreatic cancers [48]. Our results are consistent with this study. In addition, we found a strong positive linear correlation of Fp or the redox ratio with cell growth inhibition (Figure 6, R^2^ > 0.7), indicating that ORI can detect a drug sensitization effect. In comparison, ROS had an insignificant correlation with cell growth inhibition (R^2^ = 0.49, *p* = 0.12) and indicated neither the synergistic nor the addictive effect of FK866 and paclitaxel combination as observed by ORI and MTS, respectively. 

As the reported IC_50_ for HCC1806 cells for 48 h treatment is in the 1 to 5 nM range [36,37], the paclitaxel-induced growth inhibitions in the range of 1 to 20 nM we observed were not quite as significant as they would be expected for the low IC_50_, although the degree of the growth inhibition in our MTS readouts did increase with the drug’s concentration (Figure 5A). The seeding density we chose was in the linear range of this cell line for the MTS assay. It is unclear why we still observed a high viability at 20 nM. Several possibilities, such as paclitaxel degradation in alkaline medium, drug purity, and cell line variations across laboratories might account for this observation. However, since our purpose here was to investigate the correlation between the redox imaging measurements and the MTS readouts, the absolute accuracy of paclitaxel concentration is not as important as that for the determination of IC_50_. In future studies, we can determine the IC_50_ under the experimental conditions.

ORI has been used as a label-free imaging tool to study the drug response of cancer cells. It is known that metabolic changes precede the morphological manifestation of cell death and that early redox response can predict later apoptotic changes [31,32]. In the future, we may study more TNBC cell lines for their redox response profiles at various time points and correlate redox changes with various endpoints, e.g., growth inhibition, cell migration and invasion, and cell kill to gain a more holistic metabolic analysis of treatment response.

## 4. Materials and Methods

### 4.1. Cell Culture and Drug Treatments

Breast cancer HCC1806 cells and MDA-MB-231 cells (ATCC, Manassas, VA, USA) were maintained in T-25 flasks (Thermo Fisher Scientific, Waltham, MA, USA) with Roswell Park Memorial Institute 1640 Medium (RPMI 1640, Gibco Cat # 11875085, Thermo Fisher Scientific, Waltham, MA, USA) supplemented with 10% Fetal Bovine Serum (FBS) (MilliporeSigma, St. Louis, MO, USA). The cells were incubated at 37 °C with 5% CO_2_ and passaged near 80% confluence using 0.25% trypsin-EDTA (Thermo Fisher Scientific, Waltham, MA, USA). 

Dihydroethidium (DHE) and all drugs were purchased from Sigma-Aldrich (MilliporeSigma, St. Louis, MO, USA), except FK866 and paclitaxel, which were purchased from LC Laboratories (Woburn, MA, USA). Nicotinamide riboside (NR) (AST-F20758, Neta Scientific Inc. Hainesport, NJ, USA) was reconstituted in deionized water at 25 mg/mL, aliquoted, and stored at −20 °C until use. All other drugs were first reconstituted in DMSO, aliquoted, and stored at either −80 or −20 °C until use.

For FK866-treated groups, FK866 was added post cell attachment to dishes (approximately 4 h after seeding) with final concentrations between 1 and 100 nM, and cells were treated for 48 h before imaging. If NR treatment occurred, NR (800 µM final) was added to cells pre-treated with FK866 for 42 h. FK866 and NR combination treatment lasted 6 h. 

For paclitaxel-treated groups, paclitaxel was added to dishes with medium red-orange in color to avoid degradation of the drug in alkaline conditions at a final concentration of 1 and 20 nM. Treatment was 48 h. 

Acute FX11 treatment (5 µM final) was approximately 10 min. Images were taken with dishes under treatment, as opposed to the case of long-term treatment(s) where images were taken without drug presence.

### 4.2. Optical Redox Imaging of Live Cells 

Cells were seeded 50,000/1 mL onto 20 mm glass-bottom dishes (Cellvis, Cat #D35-20-1.5-N, Mountain View, CA, USA) and divided into treated and control groups. Approximately one hour before imaging, cells were rinsed twice with DPBS^+^ (Dulbecco’s Phosphate-Buffered Serum with calcium and magnesium, Thermo Fisher Scientific, Waltham, MA, USA) and incubated with 1 mL Live Cell Imaging Solution (abbreviated as LCIS, Molecular Probes, Thermo Fisher Scientific, Waltham, MA, USA) supplemented with glucose (11 mM) and L-glutamine (2 mM). A Zeiss wide-field microscope (Axio Observer 7, White Plains, NY, USA) set at 37 °C was used for imaging. Using a 20 × lens (NA = 0.8), signals were collected with an image resolution of 0.29 × 0.29 μm^2^ through the following optical bandpass filters: NADH channel, excitation (Ex) 370–400 nm, emission (Em) 414–450 nm; Fp channel, Ex 450–488 nm, Em 500–530 nm; and DHE channel, Ex 540–570 nm, Em 580–610 nm. To avoid photo-bleaching, transmitting light was used to locate and focus on regions of interest. Three to five random, distinct fields of view per dish were imaged. Shading correction was done on the fly. All dishes were imaged without the presence of drug(s), except for the acute FX11 treatment.

Intracellular ROS measurements were acquired by adding dihydroethidium (DHE, 2 µM final concentration) to dishes and incubating at 37 °C protected from light for 40 min. Dishes were then rinsed once with PBS^+^ and liquid was replaced with 1 mL LCIS supplemented with 2 mM glutamine and 11.5 mM glucose for imaging. 

### 4.3. Cell Proliferation Assay 

Cell proliferation was examined by MTS (3-(4,5-dimethylthiazol-2-yl)-5-(3-carboxymethoxyphenyl)-2-(4-sulfophenyl)-2H-tetrazolium) assay (CellTiter 96^®^ Aqueous One Solution Cell Proliferation Assay, Promega, Madison, WI, USA). Briefly, HCC1806 cells were seeded at a density of 15,000 cells or 5000 cells per well in 96-well plates and incubated overnight with RPMI containing 10% FBS. The next day, the cells were treated with various concentrations of FK866 and paclitaxel or the combination of both drugs and incubated for 48 h. Thereafter, 20 μL of MTS was added to each well. The plate was incubated for 4 h and absorbance at 490 nm was measured using a plate reader (Enspire Multimode Plate Reader, model: 2300, Perkin Elmer, Washington, MA, USA). Eight technical replicate samples were prepared in each assay. The experiment was repeated 3 times.

### 4.4. Data Analysis and Statistics

Each image file was split into its separate channels using ImageJ. A custom MATLAB^®^ (Version 2019a, The MathWorks, Inc., Natick, MA, USA) program was used to quantify NADH, Fp, and ROS intensities. Redox ratio images were generated pixel-by-pixel from NADH and Fp images. The program analyzed the images through a series of steps including background removal and thresholding at a signal-to-noise ratio of 7.5, as described in detail previously [49], except that the polynomial surface fit of the background was no longer needed for removing the vignette effect, which was corrected on the fly.

The group mean was obtained by first averaging fields of view per dish, then averaging across dishes. Bar graphs grouped by treatment are displayed as the means ± standard deviations (SD). To compare three or more groups, one-way ANOVA tests followed by post-hoc Tukey’s or Dunnett’s tests to correct for multiple comparisons were used via PRISM 9 (GraphPad Software, San Diego, CA, USA). To compare two groups, unpaired *t*-tests with unequal variance were used. Significant differences are displayed as: * *p* < 0.05, ** *p* < 0.01, *** *p* < 0.001, and **** *p* < 0.0001.

## 5. Conclusions

The present study found that the optical redox imaging technique readily detects the therapeutic effects of both single treatment of FK866 and paclitaxel and their combinations on TNBC cells. Both Fp and the redox ratio correlated strongly and linearly with drug-induced growth inhibition detected by the MTS assay. The redox indices showed synergistic changes due to the combination treatment of FK866 and paclitaxel, while MTS analysis showed an additive effect. Both Fp and NADH were found to be positively correlated with drug-induced ROS levels. Drug-induced ROS levels reflected no synergistic or addictive effects from the combination treatment, and only correlated with cell growth inhibition with a weak borderline significance. Additionally, ORI resolved differences in the treatment responses between two TNBC lines. These findings indicate that ORI is valuable for the identification of treatment responses to metabolic inhibitors targeting Nampt and the sensitization effects on standard chemotherapeutic drugs for TNBC. The findings also warrant further study by testing a panel of TNBC cells of diverse metabolism with other therapeutic agents to confirm the utility of ORI as a therapeutic response biomarker for chemotherapy of TNBC.

## Data Availability

The data presented in this study are available on request from the corresponding author.

## Supplementary Materials

The following are available online at https://www.mdpi.com/article/10.3390/ijms22115563/s1.
