# Supplementary material for: Optical Redox Imaging of Treatment Responses to Nampt Inhibition and Combination Therapy in Triple-Negative Breast Cancer Cells
Source: Int J Mol Sci. 2021 May 25;22(11):5563. doi: 10.3390/ijms22115563 (PMC8197351; doi:10.3390/ijms22115563)
Supplement: Supplementary file 1 [file ijms-22-05563-s001.zip › ijms-1225382-supplementary.pdf]

Supplementary materials

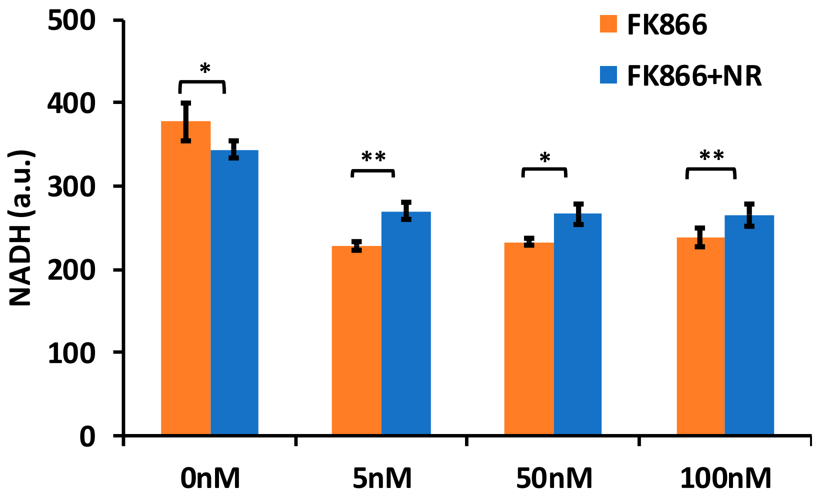

Figure S1. NR rescue effect on HCC1806 cells. Six hours NR treatment effects are nearly equivalent at various concentrations of FK866 ranging from 5 nM to100 nM. Dishes were treated for 48 hours with FK866 (or 0.1% DMSO for control represented by 0 nM) with NR (0.8 mM) added after the first 42 hours of FK866 treatment ($n \geq 3$) (Unpaired t-test, * $p < 0.05$ and ** $p < 0.01$).
